# Unmet needs in the management of psoriasis in Latin America: a systematic review^☆^

**DOI:** 10.1016/j.abd.2023.04.006

**Published:** 2023-12-06

**Authors:** Bruna Ossanai Schoenardie, Rodrigo Oliveira Almeida, Thaísa Hanemann, Arthur Ossanai Schoenardie, André Lucas Ribeiro, Juliana Catucci Boza

**Affiliations:** a Department of Dermatology, Hospital de Clínicas de Porto Alegre, Porto Alegre, RS, Brazil; b School of Medicine, Universidade Federal de Santa Maria, Santa Maria, RS, Brazil; c Department of Rheumatology, Hospital de Clínicas de Porto Alegre, Porto Alegre, RS, Brazil

**Keywords:** Delayed diagnosis of psoriasis, Health services accessibility, Latin America, Opportunistic infections

## Abstract

**Background:**

Psoriasis is a chronic, systemic inflammatory disease with a worldwide prevalence of approximately 2%. Currently, despite the difficulties faced every day by patients and physicians in low-resource countries, literature describing the exact needs of psoriasis treatment in Latin America remains scarce.

**Objective:**

To investigate the unmet needs in psoriasis treatment in Latin America.

**Methods:**

The authors conducted a systematic review following PRISMA statements in PubMed, Embase, and LILACS of studies published from January 2011 to March 2021 addressing challenges in psoriasis treatment in Latin America.

**Results:**

The search strategy identified 3,837 articles, of which 19 were included in the final analysis. Most were from Brazil (58%; n = 11), all were observational, and most were cross-sectional (84%; n = 16). Difficulties faced by psoriasis patients in Latin America included the high prevalence of opportunistic and endemic infections (42% of the studies addressed this matter; n = 8), delay in diagnosis (5%; n = 1), work productivity impairment (16%; n = 3), limited access to medication/medical care (37%; n = 7), poor adherence to treatment (5%; n = 1) and poor adherence to guidelines (11%; n = 2).

**Study limitations:**

Number and quality of studies currently available on this subject.

**Conclusions:**

Current psoriasis guidelines do not always account for epidemiological, financial, and cultural characteristics. Most studies available are from Brazil, which might not accurately represent Latin America as a whole. In a region where neglected diseases and scarce resources remain a reality, it is imperative that dermatological training be offered to primary care providers, allowing for standardized conduct and earlier diagnosis.

## Introduction

Psoriasis (Pso) is a chronic, systemic inflammatory disease presenting with cutaneous, nail and joint manifestations, affecting roughly 2% of the population worldwide.^1^, ^2^ The burden of psoriatic disease in Latin America remains largely unknown, but its prevalence is estimated at 2.1%.^3^ Pso can profoundly affect multiple dimensions of a patient’s life, including physical, emotional, occupational, social, and economic well-being.^4^ It is also associated with comorbidities such as metabolic syndrome, cardiovascular events, depression, and anxiety, further complicating disease management.^5^

Access to healthcare in many parts of Latin America remains a significant challenge, particularly for individuals residing in rural or remote areas, where delayed diagnosis is a common occurrence. The majority of these countries are still under development, and a substantial proportion of the population has limited financial resources, making it difficult to obtain even topical medications for the treatment of mild Pso. Although systemic treatments have become more accessible in recent years, the pace of these changes has not kept up with advancements in the field, leading to legal actions against the healthcare system.^6^

The higher prevalence of opportunistic and endemic diseases in Latin America, such as tuberculosis, leishmaniasis, leprosy, and hepatitis C, presents an additional challenge in the utilization of immunosuppressive therapies for moderate and severe Pso. Currently, there is a pressing need for the development of specific guidelines to address these challenges within the Latin American population.^7^

The majority of Pso studies have been conducted in developed countries, potentially failing to accurately capture the unique circumstances in Latin America due to cultural and social differences. Consequently, this systematic review seeks to assess the challenges associated with Pso management in Latin America, with the aim of identifying targeted strategies for improving patient outcomes in the region.

## Methods

The authors conducted a comprehensive systematic review to address unmet needs in the management of Pso in Latin America, adhering to the Preferred Reporting Items for Systematic Reviews and Meta-analyses (PRISMA) guidelines.^8^ This study has been registered with PROSPERO (CRD 42021241881).

Inclusion criteria encompassed original research articles examining populations of Pso patients residing in any Latin American country. The authors did not impose restrictions based on the age of study participants, the presence or absence of treatment, or the type of treatment received (topical, systemic, or phototherapy). The authors accepted all severity levels of psoriasis (mild, moderate, and severe). The main exclusion criteria were review articles and studies of patients not originating from Latin American countries.

To qualify for inclusion, studies had to evaluate regional difficulties encountered by Pso patients and healthcare providers that could adversely impact Pso diagnosis and treatment. The authors analyzed the following outcomes: limited access to treatment and judicialization; opportunistic and endemic infections; poor adherence to treatment and disease knowledge; delayed diagnosis; work productivity and socioeconomic status; and adherence to treatment guidelines.

The authors searched PubMed, Embase, and LILACS for articles published between January 2011 and March 2021. All original studies written in English, Portuguese, or Spanish were included. The authors found only one article written in French, which was excluded. The authors chose not to include congress abstracts, except in instances where the authors deemed the information to be paramount and the published abstract provided the most comprehensive information available on the subject.

Our search protocol went as follows: For Pubmed “Psoriasis”[Mesh] OR “Psoriasis” AND “Latin America”[Mesh] OR “Latin America” OR “Argentina” OR “Bolivia” OR “Brazil” OR “Brasil” OR “Chile” OR “Colombia” OR “Ecuador” OR “French Guiana” OR “Guyana Francesa” OR “Guyana” OR “Paraguay” OR “Peru” OR “Suriname” OR “Uruguay” OR “Venezuela” OR “Belize” OR “Costa Rica” OR “El Salvador” OR “Guatemala” OR “Honduras” OR “Mexico” OR “Nicaragua” OR “Panama” OR “Cuba” OR “Dominican Republic” OR “Republica Dominicana” OR “Haiti” OR “Guadeloupe” OR “Martinique” OR “Puerto Rico” OR “Saint-Barthélemy” OR “Saint-Martin” OR “Guadalupe” OR “Martinica” OR “San Bartolome” OR “San Martin” OR “Guyane francaise”. For Embase: ‘psoriasis’/exp OR ‘psoriasis’ AND ‘South and Central America’/exp OR ‘South America’ OR ‘Central America’ OR ‘Latin America’ OR ‘Argentina’ OR ‘Bolivia’ OR ‘Brazil’ OR ‘Brasil’ OR ‘Chile’ OR ‘Colombia’ OR ‘Ecuador’ OR ‘French Guiana’ OR ‘Guyana Francesa’ OR ‘Guyana’ OR ‘Paraguay’ OR ‘Peru’ OR ‘Suriname’ OR ‘Uruguay’ OR ‘Venezuela’ OR ‘Belize’ OR ‘Costa Rica’ OR ‘El Salvador’ OR ‘Guatemala’ OR ‘Honduras’ OR ‘Mexico’ OR ‘Nicaragua’ OR ‘Panama’ OR ‘Cuba’ OR ‘Dominican Republic’ OR ‘Republica Dominicana’ OR ‘Haiti’ OR ‘Guadeloupe’ OR ‘Martinique’ OR ‘Puerto Rico’ OR ‘Saint-Barthélemy’ OR ‘Saint-Martin’ OR ‘Guadalupe’ OR ‘Martinica’ OR ‘San Bartolome’ OR ‘San Martin’ OR ‘Guyane Francaise. For LILACS: “psoriase” OR “psoríase” OR “psoriasis”. The authors also added a filter for research on human beings on all three websites.

Article selection was conducted using the Rayyan QCRI tool.^9^ Abstracts were independently analyzed by two separate researchers and, when necessary, the full text was also evaluated. Disagreements were settled by consensus between the two researchers.

Data extraction was performed by another pair of independent researchers, with discrepancies resolved by consensus. All articles were appraised for risk of bias according to the Joanna Briggs Institute critical appraisal tools.^10^, ^11^, ^12^, ^13^ Risk of bias determination was carried out by two independent researchers and any discrepancies were resolved through consensus.

In addition to data relating to the study outcomes, the authors extracted the following data from the articles: general study characteristics (i.e., year of publication, country of origin), study design, financing (public, private or mixed), sample size, and demographic data.

## Results

**Figure 1:**
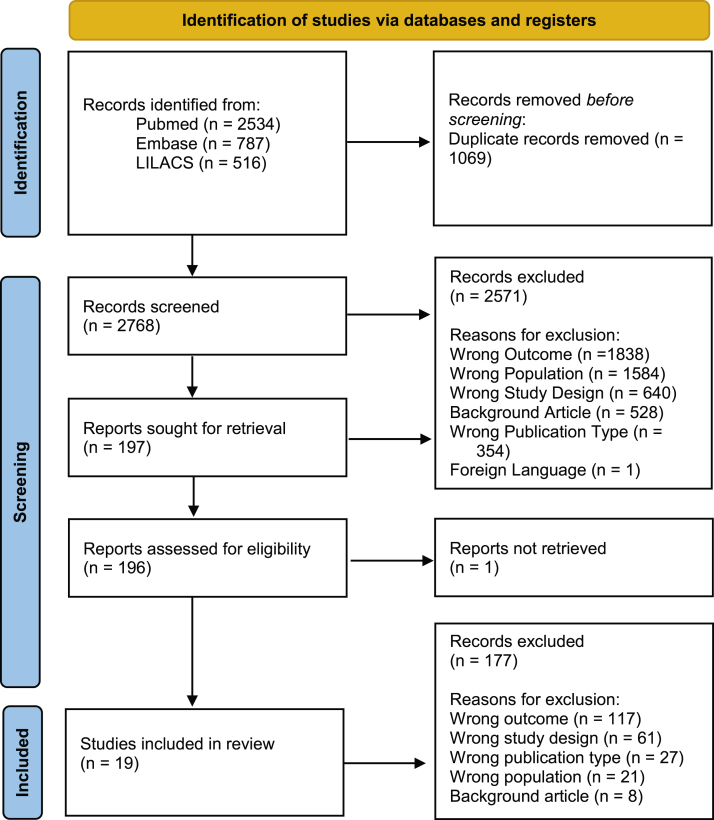
**PRISMA-style flow diagram.** Steps in article selection for inclusion in the review.

The initial search found 3,837 articles, of which 19 were ultimately included in the final analysis. The majority of articles originated from Brazil (n = 11). The reasons for article exclusion from our review were the following: wrong outcome (n = 1838), wrong population (wrong country or wrong disease; n = 1584), wrong study design (n = 640), background article (n = 528), wrong publication type (congress abstracts; n = 354) and foreign language (n = 1). Some articles were included in multiple exclusion categories. A PRISMA-style diagram detailing each step of article selection is presented in Fig. 1, and Table 1 provides a summary of all included articles.

**Table 1:** Characteristics of included articles.

Study	Year	Sample size	Study design	Country	Financing	Main outcomes
Rada JR, et al.^18^	2020	374	CS	Venezuela	Public	Opportunistic and endemic infections
Quiroz-Vergara JC, et al.^29^	2017	100	CS	Mexico	Public	Delayed diagnosis
Andrade DL, et al.^23^	2012	140	CS	Brazil	Public	Opportunistic and endemic infections
Araujo KM, et al.^26^	2020	56	RC	Brazil	Public	Opportunistic and endemic infections
Figueroa P, et al.^19^	2018	93	CS and RC	Argentina	Public	Opportunistic and endemic infections
Kurizky PS, et al.^24^	2019	311	CS	Brazil	Public	Opportunistic and endemic infections
Lopes N, et al.^30^	2019	188	CS	Brazil	Private	Access to medication/medical care/Work productivity
Úsuga F, et al.^14^	2019	312	CS	Colombia	Public	Access to medication/medical care
Lopes N, et al.^6^	2017	188	CS	Brazil	Private	Access to medication/medical care
Lopes LC, et al.^17^	2014	203	CS	Brazil	Public	Access to medication/medical care
Ferreira CN, et al.^31^	2014	210	CS	Brazil	Private	Work productivity
Kivelevitch DN, et al.^28^	2012	176	CS	Argentina	Public	Adherence to treatment
Maza RGC^7^	2019	510.9 patient-years	MA	Latin America	Public	Opportunistic and endemic infections
Silveira MSN, et al.^33^	2014	203	CS	Brazil	Public	Access to medication/medical care/Adherence to treatment guidelines
Gonçalves LMT, et al.^21^	2019	311	CS	Brazil	Public	Opportunistic and endemic infections
DiBonaventura M, et al.^15^	2018	12000	CS	Brazil	Private	Access to medication/medical care/Work productivity
Mazzuoccoloa LD, et al.^32^	2017	221	CS	Argentina	Public	Adherence to treatment guidelines
Lopes N, et al.^16^	2017	188	CS	Brazil	Private	Access to medication/medical care
Cataño J, et al.^20^	2016	101	PC	Colombia	Public	Opportunistic and endemic infections

Characteristics of all articles included in the review.

CS, Cross-Sectional; RC, Retrospective Cohort; PC, Prospective Cohort; MA, Meta-Analysis.

The overall quality of the included studies ranged from moderate to low. Most studies did not clearly state if a sample size estimation was performed, complicating the interpretation of prevalence results. This issue is particularly prominent for descriptive studies lacking appropriate statistical analysis. Additionally, most studies did not discuss strategies for identifying or addressing confounding bias. The risk of bias assessment results can be found in Table 2.

**Table 2:** Risk of bias assessment.

Analytical cross-sectional studies	Quiroz-Vergara JC, et al.^29^	Andrade DL, et al.^23^	Kurizky PS, et al.^24^	Lopes N, et al.^30^	Ferreira CN, et al.^31^	Kivelevitch DN, et al.^28^	DiBonaventura M, et al.^15^	Mazzuoccoloa LD, et al.^32^
Were the criteria for inclusion in the sample clearly defined?								
Were the study subjects and the setting described in detail?								
Was the exposure measured in a valid and reliable way?								
Were objective, standard criteria used for measurement of the condition?								
Were confounding factors identified?								
Were strategies to deal with confounding factors stated?								
Were the outcomes measured in a valid and reliable way?								
Was appropriate statistical analysis used?								

Descriptive cross-sectional studies	Rada JR, et al.^18^	Figueroa P, et al.^19^	Úsuga F, et al.^14^	Lopes N, et al.^6^	Lopes LC, et al.^17^	Silveira MSN, et al.^33^	Gonçalves LMT, et al.^21^	Lopes N, et al.^16^
Was the sample frame appropriate to address the target population?								
Were study participants sampled in an appropriate way?								
Was the sample size adequate?								
Were the study subjects and the setting described in detail?								
Was the data analysis conducted with sufficient coverage of the identified sample?								
Were valid methods used for the identification of the condition?								
Was the condition measured in a standard, reliable way for all participants?								
Was there appropriate statistical analysis?								
Was the response rate adequate, and if not, was the low response rate managed appropriately?								

Cohort studies	Araujo KM, et al.^26^	Cataño J, et al.^20^
Were the two groups similar and recruited from the same population?		
Were the exposures measured similarly to assign people to both exposed and unexposed groups?		
Was the exposure measured in a valid and reliable way?		
Were confounding factors identified?		
Were strategies to deal with confounding factors stated?		
Were the groups/participants free of the outcome at the start of the study (or at the moment of exposure)?		
Were the outcomes measured in a valid and reliable way?		
Was the follow up time reported and sufficient to be long enough for outcomes to occur?		
Was follow up complete, and if not, were the reasons to loss to follow up described and explored?		
Were strategies to address incomplete follow up utilized?		
Was appropriate statistical analysis used?		

Meta-analysis	Maza, RGC.^7^
Is the review question clearly and explicitly stated?	
Were the inclusion criteria appropriate for the review question?	
Was the search strategy appropriate?	
Were the sources and resources used to search for studies adequate?	
Were the criteria for appraising studies appropriate?	
Was critical appraisal conducted by two or more reviewers independently?	
Were there methods to minimize errors in data extraction?	
Were the methods used to combine studies appropriate?	
Was the likelihood of publication bias assessed?	
Were recommendations for policy and/or practice supported by the reported data?	
Were the specific directives for new research appropriate?	

Critical appraisal was performed utilizing the Joanna Briggs Institute Tools^10^, ^11^, ^12^, ^13^; the appropriate checklists were applied according to study design.

Y, Yes; N, No; U, Unclear; NA, Not Applicable.

### Limited access to treatment and judicialization

Úsuga et al. investigated 312 Colombian Pso patients, reporting that 23% had not received physician guidance. Moreover, 30% of them did not have access to the Immunobiologicals (IMB) they were prescribed.^14^

DiBonaventura et al. analyzed data from Brazil’s 2012 National Health and Wellness Survey (NHWS) (n = 12,000) and found that individuals with reported Pso were more likely to have a university degree, higher annual household income, higher employment rate, private insurance, be overweight/obese and have a smoking history. Pso was moderate in 20% and severe in 5.24% of this study’s population.^15^

Similarly, a Brazilian multicenter study (n = 188) found that 34.8% of the patients reported difficulties in obtaining prescribed medications, with 12.8% resorting to judicialization to acquire treatment. The primary reasons were drug unavailability (43.1%) and financial issues (38.5%). The various means by which Pso patients obtained medications were through the Brazilian National Health System (*Sistema Único de Saúde*, SUS) and out-of-pocket (38.5%); exclusively out-of-pocket (35.8%); exclusively through SUS (19.8%) and exclusively through private health insurance (1.1%). Among the study participants, 30.5 were taking IMB.^6^ Lopes et al. suggested that psoriasis undertreatment might be a reality due to limited access to IMB.^16^

Lopes et al. studied 203 Pso patients receiving IMB through court orders in São Paulo, Brazil, from 2004 to 2010, finding that 59.5% of patients obtained the medication through the writ of mandamus, with 86.2% never attempting to obtain it from a public or private health organization before taking legal action. Most patients (69.5%) acquired IMB via SUS with a private prescription and 70.3% did not undergo follow-up examinations.^17^

### Opportunistic and endemic infections

#### Tuberculosis

Rada et al. investigated the prevalence of Latent Tuberculosis (LTBI) among 374 Venezuelan Pso patients^17^ who were candidates for IMB treatment.^18^ They found that 70.9% had a non-reactive Purified Protein Derivative (PPD) test, and 10.4% had a reaction of ≥10 mm. Figueroa et al. reported a prevalence of LTBI of 16% and a 5% per year incidence rate among 93 Argentinian patients receiving systemic treatment.^19^

In contrast, Cataño et al. studied 101 Colombian patients undergoing immunobiological treatment^19^ and discovered a high prevalence of positive PPD tests (99%).^20^ Notably, their sample comprised patients attending an infectious diseases outpatient clinic, and thus had a higher pre-test probability to have LTBI). Chest X-Rays on initial evaluation were suggestive of tuberculosis calcified granulomas in 65.3% of cases. Of the patients with a diagnosis of LTBI, 82 (81.2%) completed nine-month chemoprophylaxis with isoniazid, and 16.8% developed intolerance/toxicity. Upon follow-up, three patients developed active Tuberculosis (TB). Of those, one case presented as extrapulmonary TB. Regarding IMB therapy, two of the patients were taking etanercept and one, adalimumab.

Finally, a meta-analysis estimated the incidence of tuberculosis among Latin American Pso patients taking IMB (the patients were taking either infliximab, adalimumab, or etanercept).^7^ It included studies from Brazil, Argentina, Chile, Colombia and Mexico. The reported TB mean incidence was 636 in 100,000 patients (95% CI 145‒1764 per 100,000 patients/year). This incidence rate was considerably higher than expected for this population in 2016 (41 cases per 100,000 patients). LTBI incidence varied from 18.8%‒100% (three studies).

#### Leprosy

Gonçalves et al. studied the prevalence of Mycobacterium leprae DNA in Pso and/or Psoriatic Arthritis (PsA) outpatients at a Brazilian university hospital in Brasília, Brazil.^21^ Brasília is located in Brazil’s Federal District, which in 2021 was classified as a moderately endemic area.^22^ The study included 311 patients, of whom 96 were taking IMB, 94 were on methotrexate (MTX), 69 were taking Non-Immunosuppressive Systemic Treatment (NIST), and 52 were controls. PCR for M. leprae was positive in five subjects (one control, one on MTX, and three on IMB). The anti-PGL1 test yielded positive results in 18 out of 70 patients (two on NIST, four on MTX, and 12 on IMB), while bacilloscopic tests were negative for all patients.

#### HCV

Andrade et al. evaluated the prevalence of Hepatitis C Virus (HCV) among 140 Pso patients in Salvador (Brazil)^22^ and found that 10 patients (7.1%) had HCV infection confirmed by PCR.^23^ The prevalence in this study was higher than the prevalence estimated for the city’s general population in the same period (1.5%; p = 0.003). In six patients, the diagnosis of Pso preceded the diagnosis of HCV infection.

#### Leishmaniasis

The prevalence of leishmaniasis among Pso patients in Brasília, Federal District of Brazil, was reported by Kurizky et al. (n = 311).^24^ Brazil’s Federal District is considered to be an endemic area for leishmaniasis.^25^ Subjects were divided into four groups: IMB (n = 96; subdivided into anti-TNF, IL-12/23, and IL-17A inhibitors groups), conventional immunosuppressors (MTX; n = 24), non-immunosuppressive treatments (n = 69; nonsteroidal anti-inflammatory, acitretin, phototherapy, and topical agents) and controls (n = 52). In the IMB group, the patients were taking the following drugs: adalimumab (n = 24), etanercept (n = 29), infliximab (n = 25), ustekinumab (n = 9), and secukinumab (n = 9). The probable positivity for leishmaniasis in their target population was set at 5%. Although no clinically active cases were detected, seven individuals tested positive by serology, thirteen by conventional PCR, and nine by real-time PCR. No significant difference was found between the three screening strategies. In the IMB group, only patients using anti-TNF had positive results (two of them were taking etanercept and one infliximab).^24^

#### Arbovirus infections

Araújo et al. followed 56 Pso patients from Rio de Janeiro, Brazil, who were taking IMB for at least 12 months, analyzing the incidence of zika, chikungunya, and dengue fever.^26^ Nineteen patients (36.5%) were taking adalimumab, 15 (28.8%) etanercept, 9 (17.3%) infliximab, 8 (15.4%) ustekinumab and 1 (1.9%) secukinumab. During the study period, six patients (10.7%) had confirmed arbovirus infections [chikungunya (n = 3), dengue (n = 2), and zika (n = 1)]. Of these patients, four [7.1%; chikungunya (n = 2), dengue (n = 1), and zika (n = 1)] experienced Pso exacerbation (p < 0.01), with three managed conservatively without discontinuing IMB therapy. The incidence rate for dengue, chikungunya, and zika in Rio de Janeiro varied during the study period (2016‒2018). In 2016 it was, respectively, 523.2/100,000 people-year, 94.9/100,000 people-year, and 432.7/100,000 people-year. However, in 2017, all indicators significantly improved. Respectively, 4.4/100,0000 people-year, 1.1/100,000 people-year and 0.3/100,000 people-year.^27^

### Poor adherence to treatment and disease knowledge

Kivelevitch et al. studied adherence to treatment among Argentinian Pso patients (n = 176) and reported that 33% of patients self-medicated, while 77% were non-adherent to treatment. The patients assessed in this sample were using topical drugs (97%) and systemic drugs 29%.^28^ The most common causes of non-adherence were lack of response to treatment (63%), clinical improvement (26%), economic factors (16%) and adverse effects (10%). When combined, the self-medication and non-adherence groups comprised 82% of the sample. Notably, 24% of patients believed Pso could be cured, and 86% stated they had not been informed about the risks of suspending or modifying treatment without supervision.

### Delayed diagnosis

Queiroz-Vergara et al. investigated the factors contributing to delayed Pso diagnosis in Mexico (n = 100).^29^ Their findings revealed that a mere 42% of patients received a diagnosis within one year of presenting symptoms and, among those, 89% were diagnosed by a dermatologist, even though the first medical appointment had been with a General Practitioner (GP) in 61% of cases. Of these patients, 31% had initiated treatment within the first year of diagnosis.

### Work productivity and socioeconomic status

Lopes et al. assessed the impact of Pso on work productivity and daily activities among 188 Brazilian patients.^30^ Presenteeism was more frequent than absenteeism, with a mean (Standard Deviation, SD) of 14.4% (5.5%) compared to 6.3% (13.8%). Presenteeism is defined as the act of attending work while ill or experiencing a medical condition that impedes full capacity on the job. They estimated that patients would need to increase working hours by approximately 5% to compensate for productivity losses due to Pso, with a mean of 4.7 hours (SD = 5.4). In contrast, Ferreira et al. found no significant differences in absenteeism, presenteeism, overall work impairment, and activity impairment across varying levels of Pso severity.^31^

DiBonaventura et al. estimated that, in Brazil, between 28% and 40% of working hours were either missed or rendered ineffective due to Pso (n = 210).^15^ Presenteeism was more frequent among Pso patients compared to patients without Pso (22.08% vs. 16.95%; p < 0.05). They estimated that this difference equates to an additional 10 days per employee per year. Activity impairment (26.52% vs. 20.97%) and the number of physician visits (5.18 vs. 4.27) were also significantly more common among Pso patients (p < 0.05). However, no differences were observed across severity levels.

### Adherence to treatment guidelines

Mazzuoccoloa et al. conducted a survey on the use of MTX for Pso treatment among Argentinian dermatologists (n = 221).^32^ They found that two-thirds of dermatologists included the PPD test and/or a chest X-Ray in their pretreatment work-up. Half of them expected a clinically significant response between weeks 4 and 6 of MTX treatment, 44% between weeks 8 and 12, and 6% after 12 weeks. Approximately 76% stated that they would consider treatment failure if no significant response was observed after 12 weeks. Concerning efficacy, 30% of Argentinian dermatologists deemed MTX ineffective. The only variable associated with suboptimal MTX use was the prescriber’s perception of its ineffectiveness (OR = 2.29; 95% CI 1.05–5.00, p = 0.037).^32^

Silveira et al. examined guideline adherence for the prescription of IMB among 203 patients suing the state of São Paulo, Brazil, from 2004 to 2011.^33^ They discovered that over 20% of patients had not used any conventional interventions prior to launching their lawsuit. Topical agents were used by 16% of patients and phototherapy by 36.9%. About 71% of patients had previously used non-immunosuppressive systemic treatment. Since Brazilian guidelines mandate the use of topical and systemic therapy before starting IMB, only 34 (16.7%) patients met the guideline requirements. All patients had visited a physician at least once a year, but 25.2% did not undergo any laboratory tests. Overall, complete adherence to guidelines was observed in 14.2% of cases. ^33^

## Discussion

### Limited access to medication and medical care

Most articles were published in Brazil before 2019 when a new Clinical Protocol and Therapeutic Guideline (PCDT) for Pso made available adalimumab, etanercept, ustekinumab and secukinumab without the need for legal action.^34^ Following this development, the number of lawsuits declined in the country.^10^ Subsequently, risankizumab was added to the PCDT.^35^

In the last decade in Brazil, most IMBs for Pso were acquired through lawsuits, leading to inadequate patient monitoring^6^, ^33^ and treatment interruption due to adverse effects. The lack of clear prescription requirements also contributes to physicians disregarding guidelines.^6^, ^33^ According to Silveira et al., over 20% of patients had not used any conventional therapy before resorting to legal action.^33^ Delays in the inclusion of drugs in the PCDT and their purchase by the healthcare system led many pharmaceutical companies to provide medication for the start of treatment, which might have contributed to an increase in legal claims for medications. Lopes et al. stated that pharmaceutical industries maintained frequent communication with the majority of the patients.^17^

Brazilian Pso patients tend to have greater education, income, and private insurance rates than controls, suggesting that they are more likely to be diagnosed due to better access to medical care. French and Italian studies suggest that lower education and income levels are associated with more severe disease, fewer medical appointments, and fewer systemic treatment prescriptions.^36^, ^37^ In the USA, younger age, lower income, and lack of insurance were associated with difficulties in acquiring IMB.^38^, ^39^ Therefore, it is reasonable to assume that Pso prevalence and treatment access in Latin America might be grossly underestimated due to socioeconomic reasons.

Lopes et al. found that 34.8% of patients reported difficulties in obtaining prescribed medications. Most prescriptions for topical drugs in Brazil, such as high-potency Topical Corticosteroids (TCS), despite being included in the PCDT, require special requisition and excessive bureaucracy, making their acquisition process time-consuming.^6^ In our practice at a tertiary public hospital in Southern Brazil, the authors often see patients purchasing TCS with their own resources or using readily available low-potency TCS, which is not adequate for Pso treatment.^40^

### Opportunistic and endemic infections

Studies from Venezuela^18^ and Argentina^19^ have reported similar rates of LTBI among Pso patients (10.4% and 16%, respectively). In Colombia,^20^ the prevalence was significantly higher at 99%. When analyzing this study’s data, however, it is crucial to consider the potential influence of selection bias. Globally, there is a wide range of regional differences, with LTBI estimates ranging from 8.3% to 86.1%.^41^, ^42^, ^43^, ^44^, ^45^, ^46^, ^47^ The data becomes even more contrasting when comparing developed and developing countries.

A Latin American meta-analysis^7^ examining Pso patients undergoing anti-TNF treatment found an incidence rate of 636 cases per 100,000 patients-year for TB, which is considerably higher than the prevalence expected for the general population during the same period. Moreover, a Colombian study^20^ reported TB diagnoses even after a nine-month chemoprophylaxis with isoniazid. Similar findings were reported in publications from Turkey,^48^ France^49^ and the USA.^45^ Consequently, it is suggested that prophylactic measures may not fully prevent TB and that periodic screening should be conducted, especially in endemic regions.

Anti-TNF agents are generally considered first-line IMB for Pso treatment due to their cost-effectiveness.^35^ However, their usage may be limited owing to the potential risk of LTBI reactivation in Pso patients, leading physicians to prefer more expensive IMB options, which subsequently increases the economic burden.^50^ Furthermore, the PPD test has been shown to have limitations, most notably its low specificity in high BCG vaccine coverage scenarios and its reliance on patient immunocompetence for reliable results. Alternative tests, such as Interferon-Gamma Release Assays (IGRA), have been reported to be more specific, but their availability remains limited due to their high cost.^51^

Regarding other neglected diseases, scientific research in the context of Pso is scarce. Studies have suggested that the use of anti-TNF may be a risk factor for leprosy or reactivation of subclinical infections, which could possibly be explained by an interference with granuloma formation.^52^, ^53^, ^54^ Literature also cites leishmaniasis, especially visceral cases, as a potential infectious complication of anti-TNF immunosuppression.^55^, ^56^ In the absence of specific guidelines, determining appropriate screening and therapeutic strategies can be challenging.

### Poor adherence to treatment and disease knowledge

An Argentinian study highlighted self-medication and non-adherence as significant barriers to Pso treatment in Latin America, estimating them at 82% of patients.^28^ Similarly, Zhang et al. reported that 82.4% of Chinese patients discontinued doctor-prescribed medications or resorted to self-medication.^57^ Conversely, a British review found that up to 40% of patients do not use medications as directed,^58^ while a Turkish study observed a significantly lower non-adherence rate of 44.8%.^59^

In Argentina, 86% of patients stated that they had not been informed about the risks of unsupervised treatment changes^28^; Furthermore, 24% believed Pso could be cured. A lack of disease knowledge was also reported in China,^57^ where a higher percentage of patients in the self-medication group expected a complete cure (68.9 vs. 57.9%; p < 0.001), and the consultation length related to adherence rates.

High demand for medical care often results in shorter patient-physician interactions, particularly in low-resource settings. Physicians may not allocate sufficient time to educate patients about their condition, specifically the manageable but incurable nature of Pso, which leads to unmet treatment expectations and subsequently poor adherence. This is especially important since greater treatment satisfaction has been statistically associated with improved adherence in Pso.^59^

### Delayed diagnosis

There appears to be a pressing need for enhancing dermatological training for GPs.^29^ In Mexico, 61% of patients initially consulted a GP, but 89% were ultimately diagnosed by a dermatologist. This contrasts with the situation in the UK, where 82% of Pso patients receive treatment exclusively within the primary healthcare setting.^60^

Griffiths et al. studied the impact of treatment guidelines on appropriate British referrals for specialist care.^61^ They found a significant improvement in adequate referrals in the intervention group (78%) compared to the control group (59%) (difference = 19.1%; Odds Ratio [OR = 2.47], 95% CI 1.31–4.68; ICC = 0). In Australia, GPs encounter Pso cases approximately only 10 times during their three-year training period,^62^ which is not sufficient for them to become adequately acquainted with such a complex condition. A Portuguese study reported that GPs tend not to view Pso as a systemic condition.^63^

The implementation of Pso guidelines targeting primary healthcare in Latin America could potentially shorten the time to diagnosis and better equip GPs to manage the condition, as well as alleviate the workload on tertiary centers. A cost-effective alternative would be the diffusion of telemedicine. This way, primary care providers would have the option, when necessary, of consulting with a trained dermatologist regarding treatment options and the need for referral to a tertiary center. This approach may lead to more timely and effective treatment for Pso patients, thereby improving their overall quality of life.

### Work productivity and socioeconomic status

Contrary to most studies published in other regions,^64^, ^65^, ^66^, ^67^, ^68^ Latin American literature did not find a statistically significant difference in work productivity across levels of Pso severity.^15^, ^31^ This discrepancy, however, may be attributed to the small sample sizes of these studies.

Lopes et al. was the only study that utilized the Work Productivity and Activity Impairment Questionnaire to assess work productivity loss.^30^ Their finding of a predominance of presenteeism aligned with data from a multinational study conducted by Villacorta et al.^64^ It is noteworthy that the absenteeism and presenteeism rates discovered in both studies were similar, but the mean Dermatology Life-Quality Index (DLQI) score in Lopes et al. was higher than in Villacorta et al. (mean = 7.2 [SD = 6.8]; 5.1 [95% CI 4.8‒5.4]). This could be a positive indicator since DLQI scores have been associated with worse work impairment.^64^, ^69^ Additionally, Lopes et al. included only patients with moderate or severe Pso, while Villacorta et al. had 32.6% of patients with mild Pso.^30^, ^64^ Furthermore, the unemployment rate (12.2%) was comparable to the overall Brazilian population’s unemployment rate during the same period (12.7%).^70^

Bronckers et al., conversely, found higher rates of absenteeism compared to presenteeism [mean (SD) 50% (46%); 20% (60%), respectively].^71^ This might be partially explained by the high percentage of females in their sample (70.7%).^71^, ^72^ Lopes et al. found a mean productivity loss index of 4.7% (SD = 5.4%) in the Work Limitations Questionnaire, which was lower than the one reported by Schmitt et al. (mean 7.6% [SD = 9.1%]).^30^, ^69^ Overall, work impairment due to Pso in Latin America seems to be similar to that in other regions.

### Adherence to treatment guidelines

Mazzuoccoloa et al. reported suboptimal use of MTX by 76% of dermatologists in Argentina.^32^ Comparable results were found in Holland, where 11% of dermatologists were not well-informed about guidelines. Although 80% of Dutch dermatologists use MTX in clinical practice, only 52% adhere to treatment guidelines when prescribing it.^73^ In a global survey on MTX use across 63 countries (38% European; 22.7% South American), approximately 40% of dermatologists prescribed insufficient maintenance doses of MTX,^74^ and 32.4% reported never or rarely increasing MTX dosages in patients with initial inadequate response.^74^ This could explain why 30% of Argentinian dermatologists consider MTX to be ineffective.^32^

Regarding pretreatment screening, the relatively high frequency of chest X-ray, HIV and PPD testing observed in Africa is probably due to the region’s high prevalence of HIV and tuberculosis.^74^ This may also account for the high rates of positive pre-IMB tuberculosis screening tests reported in Argentina.^32^

The limitations of the current systematic review on Pso in Latin America primarily stem from the limited availability and low quality of studies on the subject, with most research focused on Brazil, potentially hindering the generalizability of findings to the entire region. Small sample sizes in some studies, methodological differences, and variability in adherence to treatment guidelines may further impact the reliability and consistency of the results. Additionally, the lack of data on specific aspects, such as the relationship with neglected diseases, limits the conclusions that can be drawn in those areas. Despite these limitations, this review offers valuable insights and highlights areas where further research and improvements are needed.

## Conclusion

In Latin America, where access to healthcare and treatment options may be limited, the burden of Pso can be substantial. This underscores the critical necessity for early diagnosis, effective treatment, and comprehensive management of Pso to improve the quality of life and overall well-being of affected individuals.

Despite recent advances in Pso treatment accessibility, particularly in light of health policies regarding IMB, there remains a lack of objective data to assess their impact in Latin America. In a region where neglected diseases and constrained resources prevail, it is crucial to offer dermatological training to primary care providers. This approach would encourage standardized practices and enable a more prompt diagnosis of Pso.

Nonetheless, the majority of the studies included in this review are of moderate to low quality, warranting cautious interpretation of their results. Additionally, extrapolating findings from a few countries to encompass the entire continent is inherently challenging. In order to develop a more precise understanding of the current state of Pso treatment in Latin America, it is essential to conduct further well-designed studies across multiple countries. These studies would serve to fill existing knowledge gaps and guide future improvements in patient care, ultimately benefiting those affected by Pso in the region.

## Financial support

This research did not receive any specific grant from funding agencies in the public, commercial, or not-for-profit sectors.

## Authors’ contributions

Bruna Ossanai Schoenardie: The study concept and design; data collection, or analysis and interpretation of data; writing of the manuscript or critical review of important intellectual content; data collection, analysis, and interpretation; critical review of the literature; final approval of the final version of the manuscript.

Rodrigo Oliveira Almeida: Data collection, or analysis and interpretation of data; writing of the manuscript or critical review of important intellectual content; data collection, analysis, and interpretation; critical review of the literature; final approval of the final version of the manuscript.

Thaísa Hanemann: Data collection, or analysis and interpretation of data; writing of the manuscript or critical review of important intellectual content; data collection, analysis and interpretation; critical review of the literature; final approval of the final version of the manuscript.

Arthur Ossanai Schoenardie: Data collection, or analysis and interpretation of data; data collection, analysis and interpretation; critical review of the literature; final approval of the final version of the manuscript.

André Lucas Ribeiro: The study concept and design; effective participation in the research guidance; final approval of the final version of the manuscript.

Juliana Catucci Boza: The study concept and design; writing of the manuscript or critical review of important intellectual content; effective participation in the research guidance; critical review of the literature; final approval of the final version of the manuscript.

## Conflicts of interest

None declared.
